# Preparation and characterization of conjugated PVA/PANi blend films doped with functionalized graphene for thermoelectric applications

**DOI:** 10.1038/s41598-024-66691-w

**Published:** 2024-07-19

**Authors:** M. A. Morad, M. S. Abo Ghazala, M. G. El-Shaarawy, M. E. Gouda, T. Y. Elrasasi

**Affiliations:** 1 Physics Department, Faculty of Science, Menoufia University, Shebin El-Koom, Egypt; 2 Physics Department, Faculty of Science, Benha University, Benha, Egypt

**Keywords:** Materials science, Nanoscience and technology, Physics

## Abstract

Flexible nanocomposite thick films consisting of PVA_0.7_PANi_0.3_ polymer blend doped with different concentrations of nanoplatelets functionalized Graphene (NPFGx) (where x = 0, 5, 10, 15, 20, and 25 wt.%) were fabricated using the solution cast technique. Scanning electron microscopy (SEM), X-ray diffractometer (XRD), energy dispersive spectroscopy analysis (EDX), and Fourier-transform infrared spectra (FT-IR) were used to study the structure of the samples. The results showed that the ordered structure, its orientation, the PANis' well dispersion, and the electrostatic forces play a significant role in enhancing the interfaces between the polymer blend and the NPFG. Thermogravimetric analyses (TGA) and Thermoelectrical analyses (TE) showed that the PVA-PANi conducts a promised conjugated blend for thermoelectric applications. The introduction of the NPFG contents into the blend increased the TE measurements as the DC electrical conductivity **≈** 0.0114 (S cm^−1^), power factor ≈ 3.93 × 10^–3^ (W m^−1^ K^−2^), and Z.T. ≈ 8.4 × 10^–7^, for the 25 wt.% NPFG nanocomposite film. The effect of the polymers’ phonon contribution in the thermal conductivity controlling and enhancing the thermal stability of the prepared nanocomposite films.

## Introduction

An energy crisis is a significant bottleneck in the energy supply resources to the international economy. Recently, reusing waste heat has become one of the significant technical subjects for renewable energy-conversion technology for more efficient energy consumption to face this crisis in the future. In the last century, scientists focused on thermoelectric generators (TEG) relying on converting the wasted thermal energy into electrical energy, and the research works were continuously thriving. The essentials and fundamentals of thermoelectric materials are optimizing various conflicting properties to get a high *ZT* (Figure of Merit) value. Maximizing the thermoelectric Figure of merit (*ZT*) to fabricate a high-performance TE material requires a variety of conflicting properties such as high electrical conductivity as in the crystalline metal, as in glass, and high low thermal conductivity Seebeck coefficient as in the crystalline semiconductor. It is challenging to find a material that has these conflicting properties. The insulators and semiconductors have large Seebeck coefficients, but they have low carrier concentration and low electrical conductivity^1^. Applying temperature gradient to the material, the mobile charge carriers at the hot end diffuse to the cold end. The build-up of charge carriers results in an accumulated charge (negative for electrons, e– at the hot end, and positive for holes, h+ at the cold end) producing an electrostatic potential (voltage). The balance is thus reached between the chemical potential for diffusion and the electrostatic repulsion due to the charge build-up. This phenomenon is known as the Seebeck effect, where the Seebeck coefficient (S) is expressed by this formula^1^:

1 $$S= \frac{\Delta V}{\Delta T}$$

where S, $\Delta V$, and $\Delta T$ are respectively the Seebeck coefficient with unit V/K, the Seebeck voltage, and the temperature difference between sample ends.

The output power of the thermoelectrical cell is calculated by the power factor (PF) and the dimensionless Merit Figure obtained from thermoelectric cell efficiency formulas^2^:

2 $$ P.F = S^{{2}} \sigma_{{{\text{DC}}}} $$

where σDC is the DC electrical conductivity.

3 $$ ZT = (P.F){\text{K}}/\kappa $$

PF is the power factor, K is the temperature in Kelvin, and κ is the thermal conductivity.

The traditionally used TE Materials in thermoelectric generators are the metals and their alloys, for example, Ni, Cu, Bi, Al, and Sb, as well as semiconductors such as the PbTe and Bi2Te3. Regardless of whether these materials have high thermoelectric efficiency, they have a high production cost, processing, weight, and scarcity. Numerous research works have been carried out to address these issues by replacing the metals and their alloy-based thermoelectric materials with semiconductors and polymer materials^3^. Polymer TE materials have recently attracted much attention because their physical and chemical properties can be controlled to the desired properties through simple molecular modifications^4^. Consequently, organic polymers with carbon nanotubes and Graphene fillers were investigated as alternative thermoelectric materials^5^. Such as Polyacetylene, Polypyrroles [Polyanilines, Polythiophenes, Poly(2,7-carbazoles), Polyvinyl alcohol (PVA), Polyaniline (PANi)], Polythiophene(PTH), Poly(3,4 ethylenedioxythiophene): Poly(styrenesulfonate)/tosylate (PEDOT: PSS, PEDOT Tos), Polyacetylene (PA), Polypyrrole (PPY), Polycarbazoles (PC), Polyphenylenevinylene (PPV), and their derivatives^6^. The thermoelectric efficiency could be enhanced through nanostructural engineering as theoretical predictions suggestions due to increasing the electrical conductivity (through quantum dots) or decreasing the thermal conductivity (through nanowires and amorphous structures)^7,8^. Nanostructured thermoelectric devices are used today because of the difference in the electrical and thermal properties between the nanomaterials and their bulk materials.

Recently, conducting polymers drew a great emphasis to overcome the traditional role of polymers as insulating materials due to their physical properties such as electrical conductivity, optical properties, and environmental stability^9^. However, the poor solubility, thermal stability, and mechanical properties of the conducting polymers made their processing more difficult^10^. However, blending the polymers with other reactive semiconducting compounds can overcome these difficulties. Overview, there are various techniques to fabricate polymer blends such as solution blending, melt mixing, and ball milling. The poor compatibility between the blend components is the major drawback of solution blending due to the formation of aggregate phases inside the polymer matrix^11,12^. Furthermore, the fabrication of polymer nanocomposites consisting of nanosized has been drawing great attention to enormous potential applications by using polymeric initiators with functional groups inside or at the ends of the polymer chains^13,14^. In the middle of the conducting polymers, polyaniline (PANi) gains great attention because of its high electrical conductivity, cost-effectiveness, easy synthesis, and environmental stability. Nonetheless, it has poor solubility, low thermal stability, and mechanical properties. The combination with nanoparticles through the functional group of PANi. Can increase the electrical and thermal conductivities^11^.

Polyaniline is one of the most popular conductive polymers due to its high electrical and thermal properties among polymers, and it also has reversible acid–base characteristics. The PANi is a singular and hopeful conducting polymer due to its simple fabrication, low price, elevated environmental stability, and steady electrical conductivity^15^. However, the PANi has limited utilization because of its fusibility, mechanical properties, weak solubility, and processability^16^. Meanwhile, to conquer these disadvantages, PANi can be blended with water-soluble polymers such as polyvinyl alcohol (PVA), polyvinyl pyrrolidone (PVP), polyacrylic acid (PAA), and/or polystyrene sulfonic acid (PSSA)^17,18^. Numerous synthetic strategies for preparing PVA/PANi films via employing the PVA as a stabilizer, including chemical synthesis, graft polymerization, and oxidation polymerization, have been developed in recent years^18^.

The insulator semicrystalline polymer (PVA) affords processability, attractive mechanical properties, altitude chemical stability, low thermal conductivity (around 0.13 Wm^–1^ K^–1^), and perfect thermal and optical properties^19^. In addition, PVA is a water-soluble polymer due to the hydroxyl group, which is the source of hydrogen bonding, inexpensive, non-toxic, and applied in native industries^20^. So, the produced blend (PVA/PANI) has displayed perfect properties for thermoelectric applications.

Graphene is a two-dimensional crystalline material that has participated in many fields due to its unique electrical and structural properties^20^. Mainly, the fundamental thermal, mechanical, electrical, and thermoelectric properties of graphene get it to obtain a homogeneous dispersion in the polymer matrix^21^.

Herein, we report a facile method to prepare a cost-effective fabrication process of a TEG material with experimental testing of its Seebeck coefficient, thermal stability, and out-plan DC electrical conductivity consisting of a polymer blend of Polyvinyl alcohol (PVA) and Polyaniline (PANi) doped with different concentrations of Nanoplatelets functionalized graphene (NPFG). The high thermal stability and electrical conductivity of FGNP and the conductive polymer (PANi), and the low thermal conductivity of PVA and PANi may help to engineer a suitable blend of PVA/PANi doped with different concentrations of FGNP. A consolidation of PVA, PANi, and graphene nanocomposites will assist in the creation of novel materials that have improved the structural, and thermoelectric properties.

## Experimental

### Materials

PVA (polyvinyl alcohol) [(C2H4O)n (98–99% hydrolyzed, Alfa Aesar, average MW 88,000–97,000)], and NPFG (Nanoplatelets Functionalized Graphene) (Grade M with 99.5% C, 105 S cm^−1^ and 3000 W m^−1^ K^−1^) were purchased from Sigma Chemical Company in Germany, XG Sciences Company in the USA, respectively. Aniline Monomer C6H5NH2 (93.129 g mol^−1^) was purchased from QualiChem's Lab, while Hydrochloric acid (35% A.R.) and Ammonium peroxydisulfate (98% A.R.) were purchased from Merck Darmstadt Germany and LOBA CHEMIE PVT. LTD, respectively. Deionized water was used as a solvent for all studies.

### Preparation of polyaniline

The Polyaniline (PANi) powder was synthesized by chemical oxidative polymerization method^21,22^: through polymerization of aniline monomer in the presence of hydrochloric acid HCL (acts as a catalyst) and using ammonium peroxide-sulfate ((NH_4_)_2_S_2_O_8_) (acts as an oxidizing agent) where the molar ratio between the monomer and the oxidant is 4:1. In a 250 mL volumetric flask, 20 mL of aniline was dissolved in 40 mL of 1 mol L^−1^ hydrochloric acid aqueous solution. During the dissolution, the flask was kept inside a vessel containing a mixture of ice and salt at a temperature of ≈ −10 °C. Separately, 12 g of ammonium peroxide-sulfate ((NH_4_)_2_S_2_O_8_) was dissolved in 160 mL of 1 mol L^−1^ hydrochloric acid aqueous solution. The Solution of ((NH_4_)_2_S_2_O_8_) was slowly and carefully added into the flask containing the aniline acid solution for 2 h and under constant stirring. During this entire process, the reaction medium was kept at ≈ −10 °C and left at rest to polymerize for 24 h. The reaction medium changed color, going through tons of brown, blue, and green, and a solid deposit was formed at the bottom of the reaction flask. The green sediment was filtered, washed three times using 100 mL portions of 1 mol L^-1^HCl solution and similarly with acetone and dried at room temperature for 72 h. Under these conditions, the polymer obtained is in a doped state (PANi-HCl) (emeraldine salt) strongly suspended in water.

### Synthesis of (PVA_0.7_PANi_0.3_) blend

30 wt.% (0.3 g) of the prepared PANi-HCL was added to 30 mL distilled water for 6 h using stirring under room temperature and then for 1 h at room temperature using an ultrasonic probe. Besides, 70 wt.% (0.7 g) PVA was dissolved in 20 mL distilled water in another beaker at 70 °C under persistent stirring for 8 h, then left the PVA solution for 4 h to reach room temperature. PVA_0.7_PANi_0.3_ polymer blend was prepared via the casting method after PANi Solution mingled with the PVA solution (at room temperature) under continual stirring for 12 h.

### Synthesis of PVA/PANi/NPFG nanocomposite thick films

**Figure 1:**
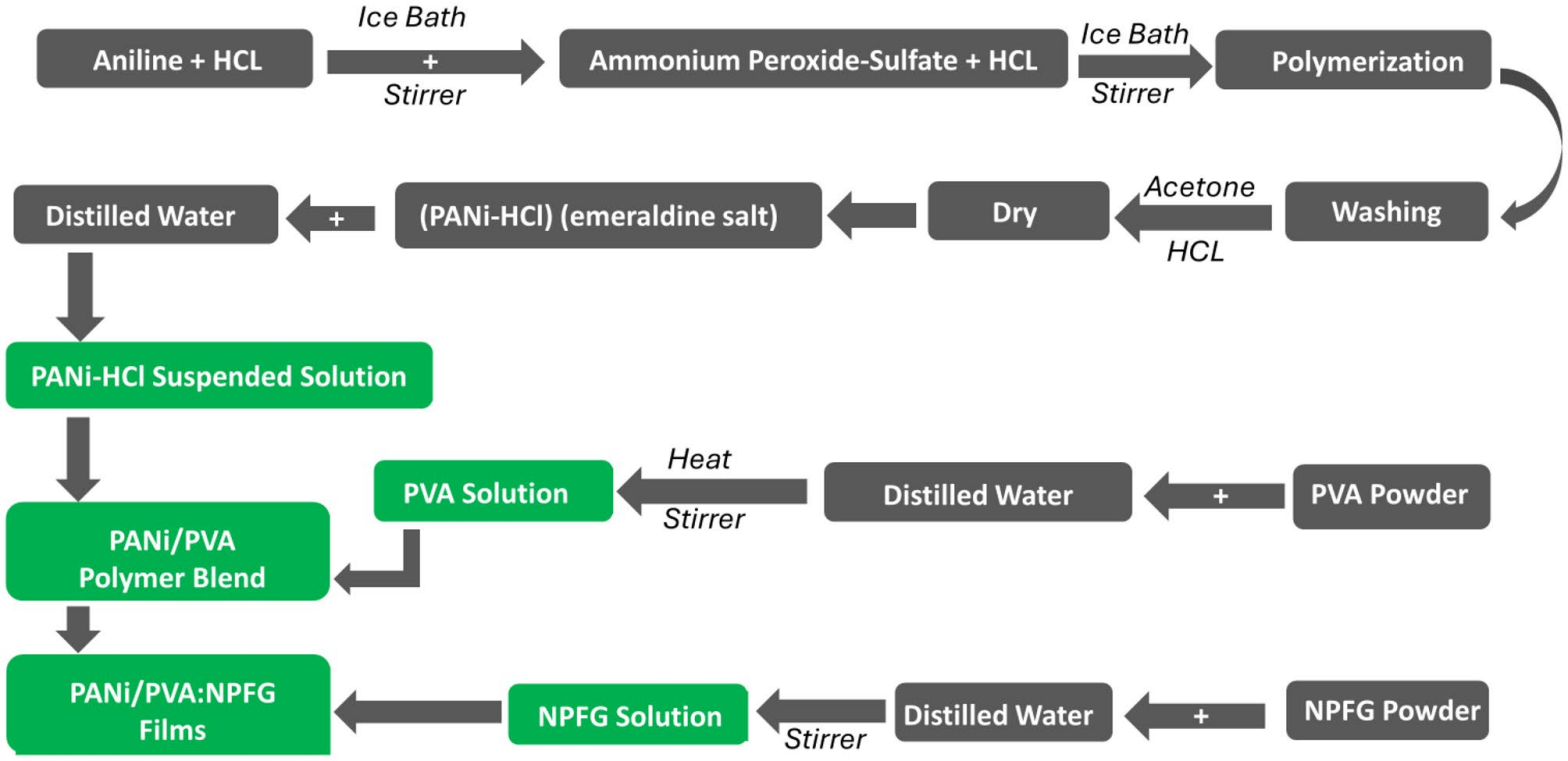
The preparation scheme of the PVA/PANi blend doped with different concentrations of NPFG.

Different concentrations of NPFG_x_ nanoparticles (x = 5, 10, 15, 20, and 25 wt.%) of the PVA_0.7_PANi_0.3_ polymer blend were dissolved in 20 mL distilled water in separated beakers at 70 °C under persistent stirring for 6 h, and then for 1 h at room temperature using an ultrasonic probe. After confirming the PVA_0.7_PANi_0.3_ blend solution, we added NPFG solutions, used the magnet stirrer at room temperature for 12 h, and then cast the film in a petri dish for three days. The schematic preparation of the nanocomposite films presented in Fig. 1.

### Characterization techniques

To study the molecular interactions of the prepared nanocomposite films, Fourier-Transform Infrared Spectra (FT-IR) scans were recorded using FT-IR spectrometer Bruker ALPHA II infrared spectrometer in the wave number range (4000–500 cm^−1^).

The structural properties of nanocomposite films were studied using XRD Rigaku Miniflex 600 X-Ray Diffractometer. The diffraction system is based on a Cu tube anode with a voltage ≈of 40 kV, current ≈30 mA, and wavelength Ka1 = 1.5418 Å has been used to calculate the crystallographic spacing utilizing the Bragg's law's Law (2d sinθ = nλ).

The surface morphology of the films was examined by analyzing the scanning electron microscopy (SEM) images. The Energy Dispersive Spectroscopy Analysis (EDX) was recorded using a scanning electron microscope (JEOL released the JCM-7000 Benchtop SEM), operated at 20 kV.

The thermal decomposition behavior of the prepared nanocomposite films was conducted by the Setaram Themys thermogravimetric analyzer at a heating rate of 10 °C min^−1^ under a nitrogen atmosphere from room temperature to 800 °C.

Films from the pristine nanocomposites with a thickness ≈ 0.2 mm, length ≈ of 30 mm and width ≈ 1.5 mm were prepared for the thermoelectrical measurements. The thermal conductivity of the prepared films was measured by Linseis LZT Laser Flash Meter—(Model LFA 1000) in a temperature range of 20 °C to 120 °C with a heating rate of 1 °C/min out of plan the films. The films' out-of-plane DC electrical conductivity and Seebeck coefficient were measured between 20 and 120 °C using the Linseis LSR-3 system with a heating rate of 1 °C/min.

## Results and discussion

### X-ray diffraction

**Figure 2:**
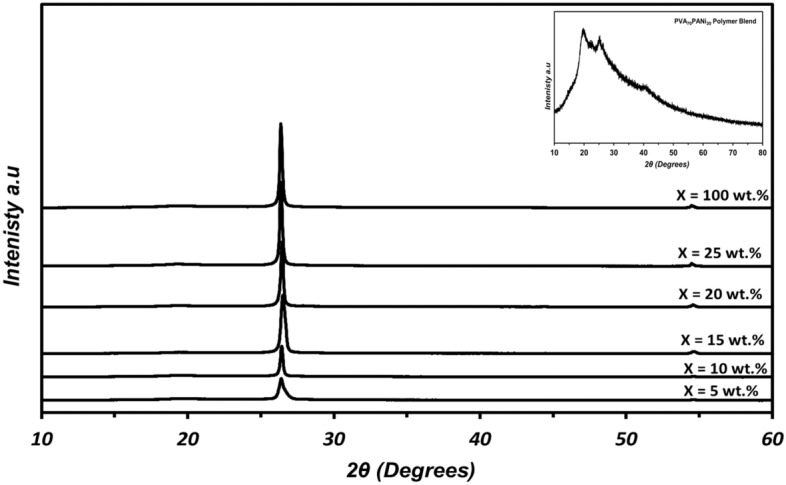
The X-ray diffraction patterns for prepared (PVA_0.7_PANi_0.3_)/NPFG_x_ nanocomposite films (where x = 5, 10, 15, 20, 25, and 100 wt. %).

The XRD patterns of the synthesized (PVA_0.7_PANi_0.3_) blend and (PVA_0.7_PANi_0.3_)/NPFGx nanocomposite films at room temperature in the scanning range 10° ≤ 2θ ≤ 60° are shown in Fig. 2. The XRD pattern of the (PVA_0.7_PANi_0.3_) polymer blend showed two humps centered at about 2θ = 20° and 25°. In particular, the peak at 2θ = 20° corresponds to a d spacing of 4.19 Å and crystal plane (101) for the host material (PVA) which confirms the distance between individual benzene rings within adjacent chains. The peak located at 2θ = 25° indicates the scattering of the PANi chains at inter-level spacing. Another small peak around 2θ = 40.0° indicates the presence of a semicrystalline structure for PVA due to its intermolecular interactions between PVA chains through hydrogen bonds^22,23^. The XRD pattern of the polymer blend film revealed the features of pure PVA but with a lower intensity of the crystal peaks. The main peak of nanoplatelets functionalized graphene (NPFG) appeared at 2θ = 26.3°corresponds to the (002) crystal reflection plane with 0.45 nm d-spacing, further additional two small peaks appeared at 2θ = 43.72° and 2θ = 54.42° correspond to the (100), (004) reflection planes with 2.1 and 1.6 nm d-spacing, respectively.

By increasing the NPFG contents in the nanocomposites, the peak becomes more intense due to increasing the crystalline character of NPFG^24^. The NPFG increases the crystalline nature of the nanocomposite films through the hydrogen bonds between the COOH, OH, and H, as well as appearing the NPFG characterized peaks at (002) reflection planes indicating the exfoliation of the NPFG^25–30^. The average crystal size (D) of the nanocomposite films was determined from the (002) reflection using the well-known Scherrer equation^25^:

4 $$D= \frac{0.9\lambda }{\beta Cos\theta }$$

5 $$\upvarepsilon =\frac{\beta }{4Tan(\theta )}$$

Where 0.9 is the Scherrer constant, λ is the X-ray wavelength, B is the width of the pure diffraction profile, and θ is the incident angle of the X-ray.

The estimated particle size of the investigated films is listed in Table 1; the estimated particle sizes of the NPFG doped films increased with increasing the NPFG content up to 15 wt.% nanocomposite, and slightly decreased on the 20 wt.%, and the 25 wt.% nanocomposites. This can be attributed to the crystallinity nature of the functionalized graphene nanoplatelets (the agglomerations of the NPFG particle on the blend)^31,32^.

**Table 1:** The structure parameters for the prepared samples: (PVA_0.7_PANi_0.3_)/NPFG_x_ nanocomposite films (where x = 5, 10, 15, 20, 25, and 100 wt. %).

NPFG content (wt. %)	XDR parameters					
	0 wt.%	5 wt.%	10 wt.%	15 wt.%	20 wt.%	25 wt.%
Particle size (nm)	–	16.14	25.14	44.21	42.06	32.72
Lattice strain (Ɛ)	–	0.40	0.23	0.15	0.14	0.16

### FT-IR spectra

**Figure 3:**
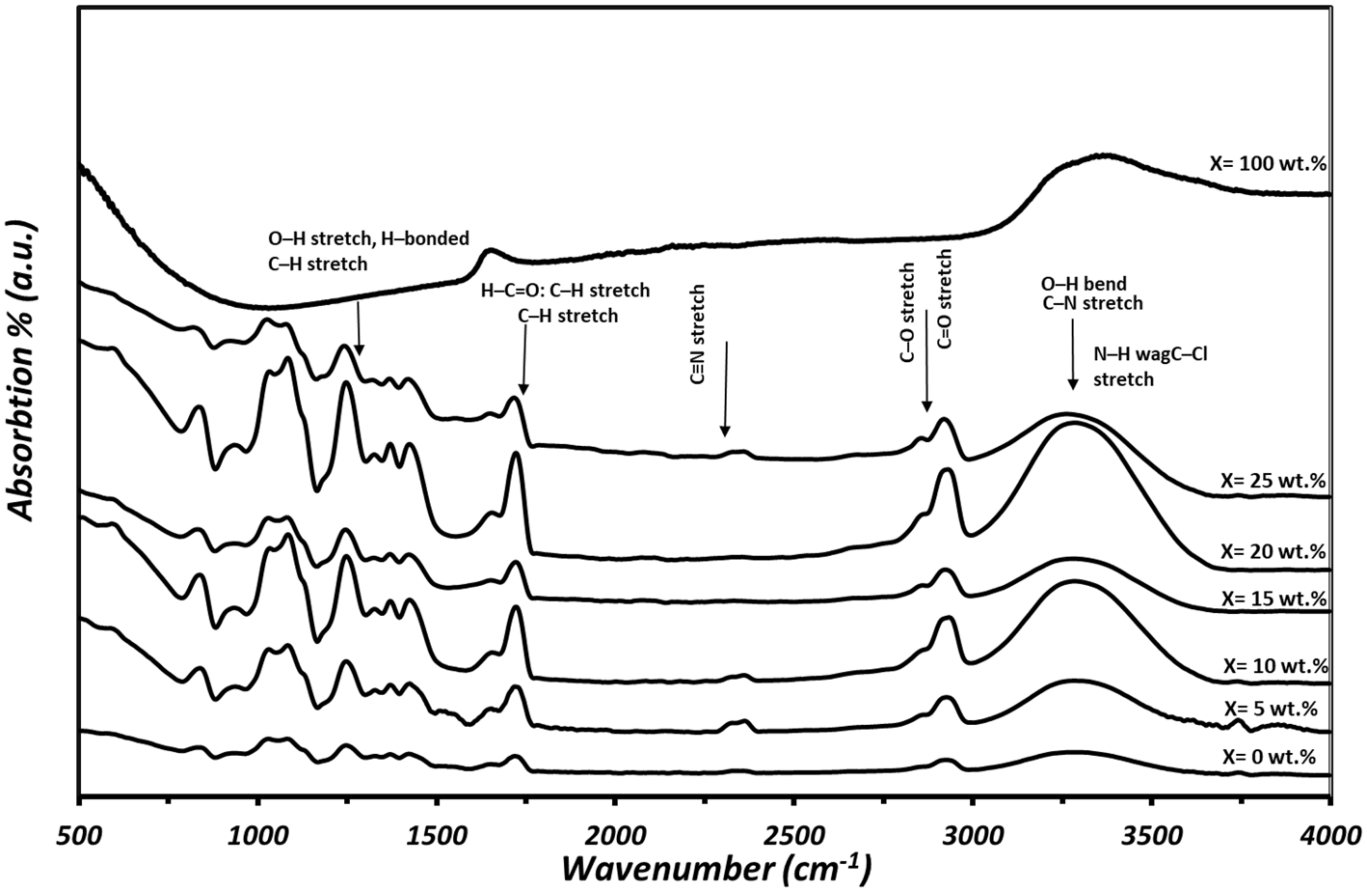
FTIR spectra patterns for polymer blend and nanocomposite films.

Fourier transform infrared spectroscopy was used to investigate the functional groups and identify the interactions of the prepared nanocomposite films. The FT-IR spectra of the tested materials are shown in Fig. 3. Firstly, the nanoplatelets functionalized graphene spectrum characterized significant bands indicating the presence of functional groups on the NPFG surfaces. The band located at ≈ 1650 cm^−1^ was observed due to the vibration of water molecules, carbonyl, and carboxyl groups. The observed wide band at 3390 cm^−1^ corresponds to the strong vibrations of the hydroxyl group^33^.

Some quite broad and severally overlapped characteristic bands of the (PVA_0.7_PANi_0.3_) film appeared on the spectrum range 1600–600 cm^−1^ which can be attributed to the polymer formation^34^. The appearance of a peak near 825 cm^−1^ corresponds to N–H, C–CL stretch, and the π to the localized polaron band transition^35^. As well as the C–C stretching vibration band appeared at 864 cm^−1^. The absorption band at ≈ 1081 cm^−1^ is attributed to the C–O stretching vibration band. The intensity of the C–O band was a measure of the degree of crystallinity of polymers^36^. A band located at ≈ 1255 cm^−1^ occurs due to C–C stretching. The absorption bands at ≈ 1498 and 1440 cm^−1^ are assigned to CH2 bending vibration^36^. A sharp peak at 1717.87 cm^−1^ was assigned to the C=C stretching of the quinoid ring (N=Q=N)), the OH bending peak appeared at ≈1640 cm^−1^, the peak appeared at 1470 cm^−1^ denotes the C=C stretching vibration of the benzenoid ring (N–B–N)), at the wavenumber ≈1240 cm^−1^, the C–N stretching of the secondary aromatic ring appeared, where the aromatic C–H in-plane bending vibrations was shown at ≈1100 cm^−1^, and at 804.32 cm^−1^ the aromatic C–H out-of-plane bending vibrations is obtained in the spectrum. These peaks match well with those mentioned in previous works^35–37^. The band at ≈ 1753 cm^−1^ corresponds to symmetric stretching vibrations of the C=O bond. As well as a broad peak centered at 3445.7 cm^−1^ corresponds mainly to the O–H stretching vibrations of hydroxyl groups of the blend. The OH and C–H stretching vibrations of secondary amine indicate the well cross-linking of the PANi in the blend. Furthermore, the polymer blend shows similar infrared responses on both constituents of the film, indicating a good homogeneity of PANi in the blend.

With the introduction of the NPFG nanofillers, the intensity of the absorption band at 3380 cm^−1^ increased and shifted slightly to lower wavenumbers with increasing the NPFG contents. The symmetric and anti-symmetric CH_2_ bands at 2929–2860 cm^−1^ are also shifted and slightly lose their intensity with increasing nanofiller concentrations in the polymer blend^38^. These results suggest that hydrogen bonding exists between the polymer blend and functionalized graphene, and crystallinity increases with NPFG introduction, which confirms the XRD results^36,39^.

### SEM morphology and energy-dispersive X-ray (EDX)

**Figure 4:**
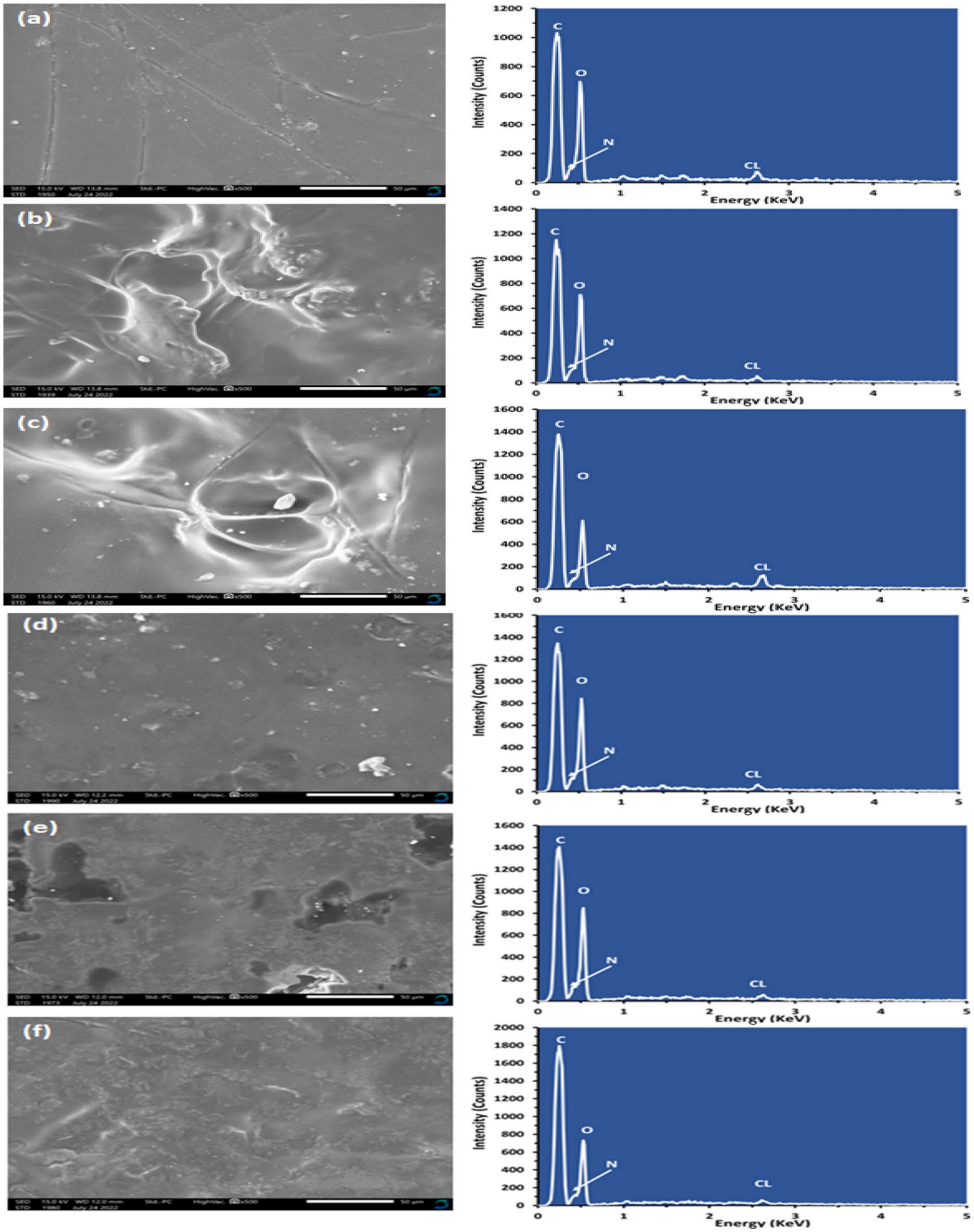
SEM images of (PVA_0.7_PANi_0.3_)/NPFG_x_ nanocomposite films (where (**a**) x = 0, (**b**) x = 5, (**c**) x = 10, (**d**) x = 15, (**e**) x = 20, and (**d**) x = 25 wt.%).

A scanning Electron Microscope was utilized to examine the morphology of the (PVA_0.7_PANi_0.3_)/NPFG_x_ films. Figure 4a–f demonstrates the surface morphology of the tested films at a magnification of  500×. The image of the polymer blend in Fig. 4a shows the film's slightly porous spongy morphology, which provides a large surface area. The influence of the aniline concentration in the film was also exhibited, as some cracks or unevenness were developed due to the incorporation or the crosslinking of PANi into the PVA. The aniline in the blend forms a partial agglomeration of coral-like PANi Particles to form clusters of flakes in the lattice structure, indicating the microcrystalline nature of PANi. The formation of PANi clusters within the polymer blend could be attributed to the hydrophobic nature of PANi molecules, which may aggregate due to hydrophobic dispersion force in a rod-shaped form. Attentively, there are no separate domains for conducting and insulating components visible, which conduct interlayered association of the blend's components and a unique chemical homogeneity of the prepared film. The SEM image shown in Fig. 4a confirmed the formation of a homogenous interpenetrating network of polymer blends^34,40^.

The images of the (PVA_0.7_PANi_0.3_)/NPFG_x_ nanocomposite films are shown in Fig. 4b–f. As the NPFG content increased, the NPFG in the nanocomposite films became more. At the low NPFG contents for 5 wt.%, 10 wt.%, and 15 wt.% nanocomposite films, the polymer chains were intertwined and electrostatically bonded. At the same time, a few agglomerations of the NPFGs were observed. The dispersion of the NPFG appeared well at the 20 wt.% and 25 wt.% nanocomposite films. As expected, the 20 wt. %, and 25 wt.% NPFG nanocomposite films exhibited highly ordered NPFG, and polymer intercalated. The potential H-bonding between NPFG and the blend enhanced the nanocomposite films' interfacial adhesion and mechanical properties, confirming the FT-IR Spectra and the XRD analysis^41^.

The elemental composition of the prepared films was analyzed by energy-dispersive X-ray (EDX) analysis. The EDX results were measured based on the SEM images of  1000× magnification. The EDX spectrum disclosed only the existence of carbon, oxygen, nitrogen, and chlorine, and no impurities could be observed in the spectrum of samples, as shown in Table 2.

**Table 2:** EDX analysis Atoms percentage and elements weight for the (PVA_0.7_PANi_0.3_)/NPFG_x_ nanocomposite films (where x = 5, 10, 15, 20, and 25 wt. %).

Elements	0 wt.%	5 wt.%	10 wt.%	15 wt.%	20 wt.%	25 wt.%
	Mass (%)	Mass (%)	Mass (%)	Mass (%)	Mass (%)	Mass (%)
C	31.38	32.6	33.09	35.98	40.93	45.88
N	10.53	9.01	9.86	7.98	7.03	5.23
O	56.51	57.43	56.22	54.96	51.14	46.5
Cl	1.59	0.96	0.83	1.08	0.9	2.4

### Thermogravimetric analysis (TGA)

**Figure 5:**
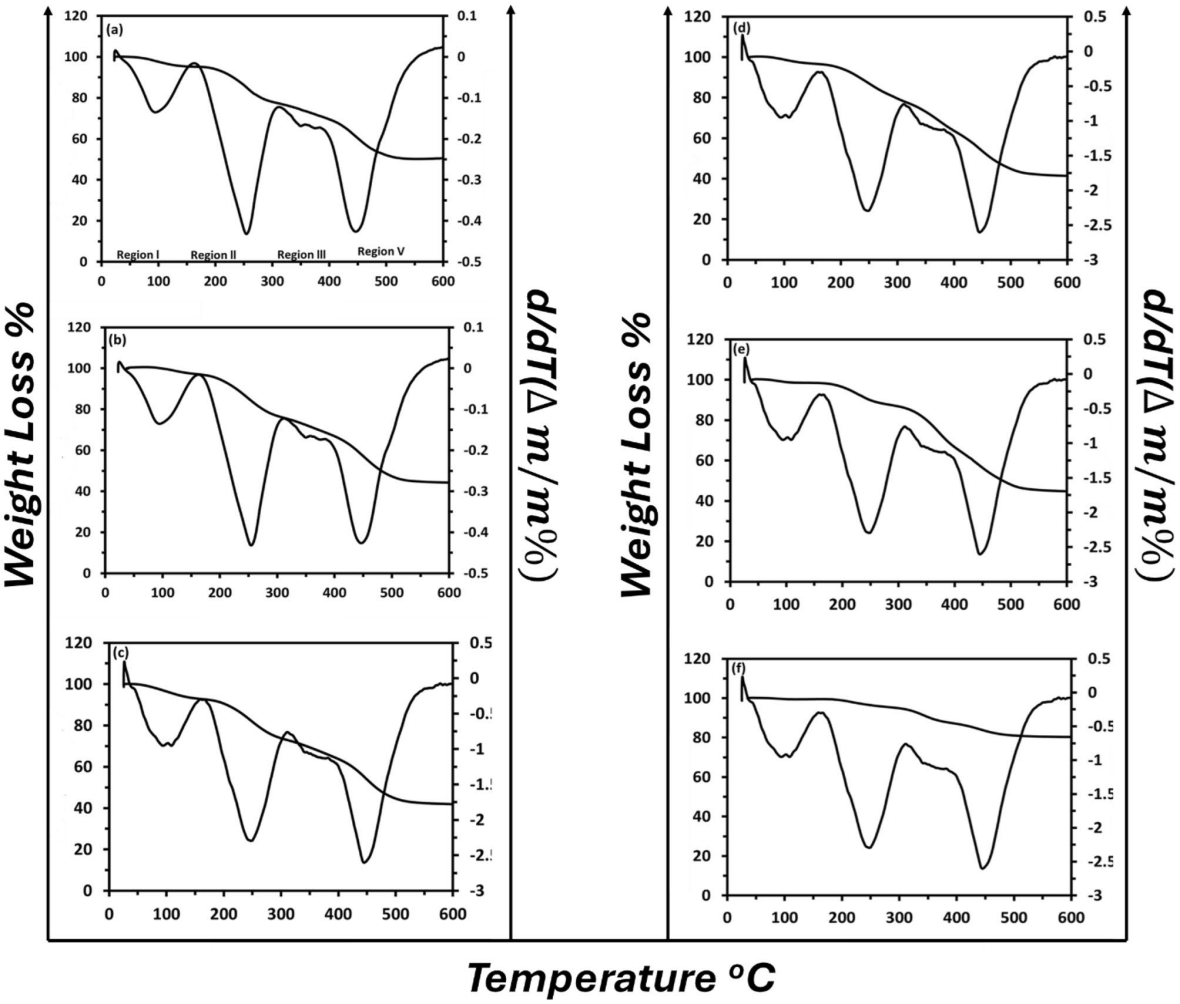
The TGA thermograms weight loss and its derivative versus temperature for the (PVA_0.7_PANi_0.3_)/NPFG_x_ nanocomposite films (where (**a**) x = 0, (**b**) x = 5, (**c**) x = 10, (**d**) x = 15, (**e**) x = 20, and (**d**) x = 25 wt. %).

Thermogravimetric analysis (TGA) is the most important method for studying and investigating the nanocomposites' thermal stability. In Fig. 5, thermogravimetric patterns and their derivatives were plotted for the prepared films by heating the samples from room temperature up to 600 °C at a heating rate of 10 °C min^−1^ under a nitrogen atmosphere. Four thermal weight loss regions can be observed: 30–150 °C, 150–300 °C, 300–400 °C, and 400–525 °C. The first weight loss corresponds to a loss of moisture, dehydration in the nanocomposite samples, and hydrochlorination (HCl), which is chemically combined in the composition of the polymer blend^42^. With increasing the temperature gradually, the adsorbed water (including the chemisorbed or physically absorbed water molecules) is released, and the weight loss percentages varied ≈ from 7.3 and 0.4% for the 0 wt.%, 25 wt.% NPFG films at T = 150 °C, respectively. The rapid weight losses that occur in the second and third regions (over 150–400 °C) can be attributed to the melting of the blend accompanied by some vapor loss, as well as the thermal degradation (first degradation process) of polymer blend intermolecular hydrogen bonding on the nanocomposite films^43^. The attributions of the decomposition of the blend with gases released like the carbon dioxide (CO_2_), melting, and partial thermal decomposition of NPFG dominate the fourth weight loss region exhibiting the lowest weight loss, about 17.3% for the 25 wt.% NPFG film^44^. The thermal parameters of all the nanocomposite films are reported in Table 3.

**Table 3:** Thermal parameters for (PVA_0.7_PANi_0.3_)/NPFG_x_ films (x = 5, 10, 15, 20, and 25 wt.%).

NPFG wt.%	TGA results			
	Peaks temperature ^o^C			Residual weight (mg) at 525 °C (wt.%)
	T_2_	T_3_	T_4_	
0 wt.%	255	340	410	50.6
5 wt.%	250	335	415	45.4
10 wt.%	252	350	418	43.1
15 wt.%	255	380	420	42.8
20 wt.%	248	375	435	46.1
25 wt.%	230	345	440	80.7

The derivative thermogravimetric analysis (DrTGA) is more helpful in ensuring the above illustration, as presented in Fig. 5. For the polymer blend (0 wt.%) sample, the DrTGA peaks centered at 80 °C, 270 °C, and 420 °C corresponded to water dehydration, melting, and the decomposition (first and second degradation) of the polymer blend, respectively. These peaks were shifted towards higher temperatures for the samples doped with NPFG. This means that with Increasing the NPFG content, the decomposition temperature increases, i.e., increasing the samples' thermal stability.

Upon the above considerations, the functionalized graphene nanoplatelets improved the thermal stability of the polymer blend, which may result from the increment of the crystallinity of the nanocomposite films that happened by the NPFG introduction^39,45^.

### DC electrical conductivity

**Figure 6:**
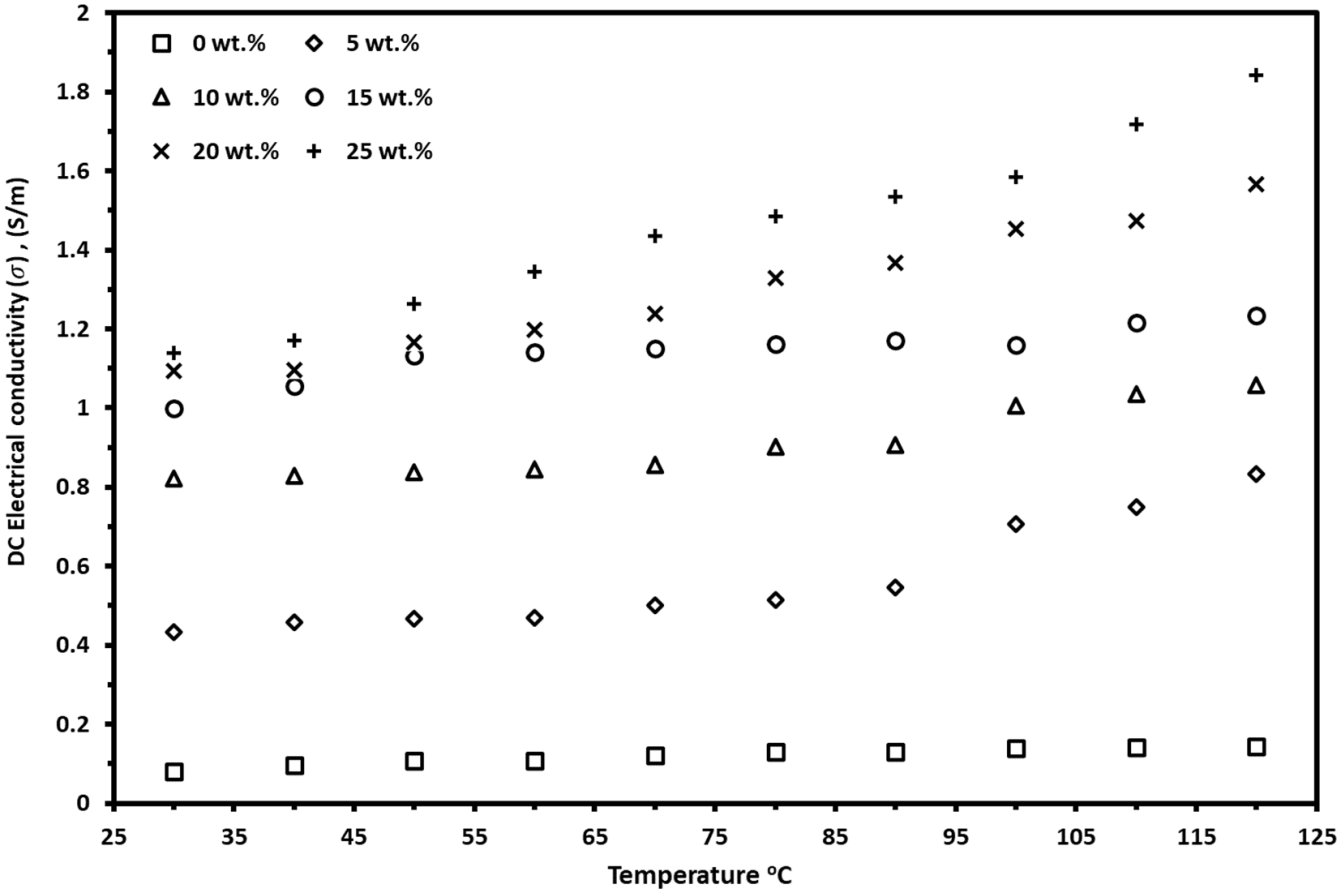
The temperature dependence of DC conductivity for the prepared nanocomposite films.

The temperature dependence of DC conductivity, σ_DC_, was carried out for (*PVA*_70%_*/PANI*_30%_)*/NPFG*_*x*_ thick films in the temperature range 303–393 K is depicted in Fig. 6. The conductivity values of all investigated nanocomposite thick films are higher than those found for pure *(PVA*_70%_*/PANI*_30%_)* polymer blends*. The Figure also shows that the σ_DC_ increases steadily with temperature showing semiconductor behavior which can be attributed to the aggravated thermal motion of the molecules which increased the carrier concentration with the elevated temperature^34^.

More looking in Fig. 6, the DC conductivity—composition dependence at room temperature shows that the σ_DC_ increases with increasing the NPFG content in the polymer blend attaining an almost equal value at higher concentrations of NPFG, which is attributed to the saturation of the charge carriers. The electrical conductivity increases from 0.08 to 1.0 S m^−1^ at room temperature for pure polymer blend and 15 wt.% NPFG nanocomposite film, respectively as mentioned in Table 4. This increment is a result of the increase of the charge carrier concentration with the NPFG concentration increasing^46^. The well-uniformly NPFG dispersion in the blend matrix forms a superior conductive network and simultaneously increases the contact area between the polymers blend and NPFG through the strong hydrogen bonds interaction as well as the π–π-bonded surface which leads to a higher conjugated system as confirmed by the FTIR and XRD results^47,48^.

**Table 4:** Electrical thermoelectrical parameters for the (PVA_0.7_PANi_0.3_)/NPFG_x_ nanocomposite films at room temperature.

NPFG (wt.%)	At room temperature								
	${\upsigma }_{\text{DC}}$ (S m^−1^)	T_0_ (K)	N(E_f_) (eV^−1^ cm^−1^)	R (A^0^)	W (eV)	S (µV K^−1^)	P.F (µW m^−1^ K^2^)	ZT	K (W m^−1^ K^−1^)
0 wt.%	0.08	4.06 × 10^6^	3.66 × 10^20^	40.46	0.0697	−18.94	2.84 × 10^–5^	2.4 × 10^–8^	0.362
5 wt.%	0.43	6.88 × 10^5^	2.16 × 10^21^	25.95	0.0447	−21.47	1.99 × 10^–4^	1.5 × 10^–7^	0.409
10 wt.%	0.82	4.93 × 10^5^	3.01 × 10^21^	23.88	0.0412	−27.05	6.02 × 10^–4^	3.7 × 10^–7^	0.491
15 wt.%	1.00	1.59 × 10^5^	9.34 × 10^21^	17.99	0.031	−29.48	8.68 × 10^–4^	4.3 × 10^–7^	0.613
20 wt.%	1.09	1.25 × 10^6^	1.18 × 10^21^	30.16	0.052	−45.70	2.28 × 10^–3^	8.5 × 10^–7^	0.818
25 wt.%	1.14	2.48 × 10^6^	5.98 × 10^20^	35.77	0.062	−50.70	2.93 × 10^–3^	8.4 × 10^–7^	1.063

To investigate the conduction mechanism, several models are applied to the DC electrical conductivity experimental data at the temperature range. We found that the best fitting for the experimental data was shown by Greave's model. According to the Greaves model, the conductivity is attributed to the hopping of charge carriers in three-dimensional between localized states at the Fermi level. It can be expressed by^48,49^:

6 $${\sigma }_{DC}\left(T\right)= {\sigma }_{0}{T}^{-1/2}exp{(-T/{T}_{0})}^{1/4}$$

Where ${\sigma }_{0}$ is a constant independent of temperature and *T*_*o*_ is the Mott characteristics temperature which has the formula:

7 $${T}_{0}= 16/(KN\left({E}_{f }\right){L}^{3})$$

Where *L* is the localization length and *N*(*E*_*f*_) is the density of states and is estimated by assuming *L* value of 5 Å for the blend. The calculated values of *N*(*E*_*f*_) for prepared nanocomposite thick films increased with the NPFG content increasing, up to x = 15 wt.%, then it slightly decreased at higher concentrations. By increasing the NPFG content, the charge carrier’s concentration increases which increases the density of states up to the x = 15 wt.% nanocomposite film. At the higher NPFG concentration, the estimated values of the *N*(*E*_*f*_) decrease as the NPFG content increases. This is due to the increasing in the crystallinity of the samples with increasing the concentration of the NPFG as confirmed by the XRD diffraction.

The mean hopping distance *R*_*hopp*_ between two adjacent sites through a barrier height *W*_*hopp*_ is calculated by the following equations^49^;

8 $${R}_{hopp}=(3/8) {L({T}_{0}/T)}^{1/4}$$

9 $${W}_{hopp}=(1/4) {KT({T}_{0}/T)}^{1/4}$$

The *R*_*hopp*_ and *W*_*hopp*_ for (PVA_0.7_PANi_0.3_)*/NPFG*_*x*_ nanocomposite films decreased with increasing NPFG content up to the x = 15 wt.% film. Then it increased showing the opposite trend of the density of states because of the changes in the crystallinity, the charge carriers, and mobility as listed in Table 4.

### Thermoelectric measurements

**Figure 7:**
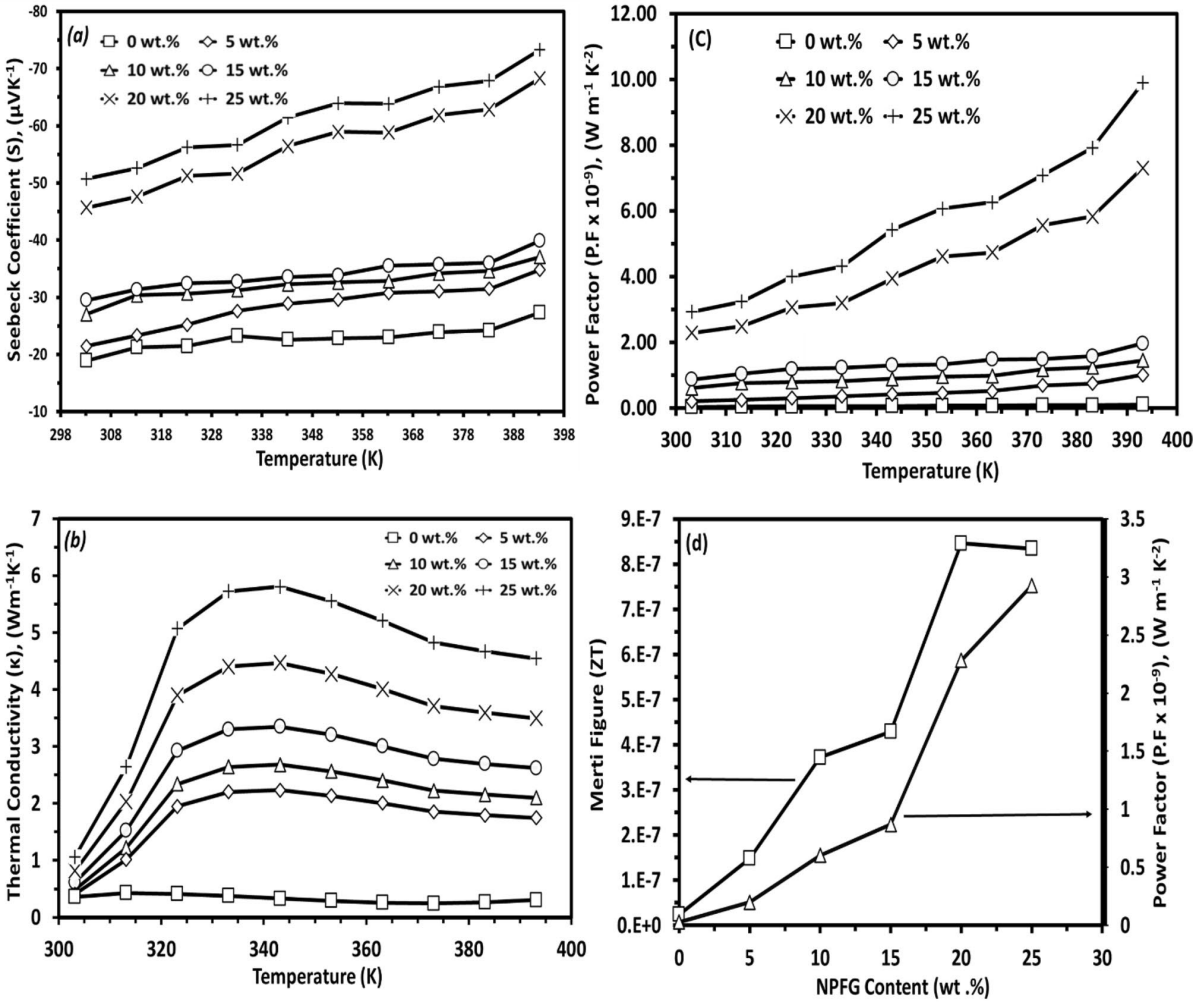
The temperature dependence of; (**a**) Seebeck coefficient, (**b**) thermal conductivity, merit figure, and (**d**) shows the composition dependence of the power factor and merit figure for the prepared nanocomposite films.

The temperature dependence of Seebeck coefficient for all the papered samples is depicted in Fig. 7a. All the prepared films are n-type semiconductors due to the negative Seebeck coefficient. However, the negative (−) sign identifies the type of charge carrier, not the Seebeck coefficient value^50^. The Figure shows that Seebeck coefficient increases steadily with the temperature increasing for all the prepared samples.

At room temperature, with the content of NPFG increasing, the Seebeck coefficient of all the nanocomposite films is remarkably increased. The Seebeck coefficient of the composite increased from −18.9 to −50.7 μV/K with increasing the GNP content from 0 to 25 wt.%, respectively. The same behavior was observed in some published data for carbon nanotubes^51^. This behavior could be attributed to the introduction of higher densities of interfaces into the system by increasing the NPFG content. As a result, additional energy barriers arise. Part of the carriers will be blocked by the energy barriers and filtered out due to the low energy, which leads to the carriers of average energy jumping over the barriers being enhanced, thereby increasing the Seebeck coefficient.

It is clear in Fig. 7b, that for all the NPFG nanocomposite films, the thermal conductivity increased with the temperature rising to a certain temperature, and then, it decreased with the temperature increase. At room temperature, nanocomposite films' thermal conductivity is slightly increased with increasing the NPFG content to the polymers' blend. Generally, the carrier mobility of conductive polymers is much lower than that of metals due to the structure of conductive polymers. The charge carrier contribution to the thermal conductivity is generally small for conductive polymers, while the phonon contribution is dominant^46,52^. Numerous nanocomposite interfaces may act as phonons' effective scattering centers. Phonons are highly scattered. Thermal transport in films is impeded, while a hopping transport mechanism can maintain the electrical conductivity. This is consistent with the low thermal and high electrical conductivity obtained experimentally for (PVA_0.7_PANi_0.3_)/NPFG_x_ nanocomposite films. The power factor temperature dependence showed the behavior of the Seebeck coefficient which is a normal mathematically of multiplying the Seebeck coefficient and the DC electrical conductivity, as conducted in Fig. 7c. Figure 7d shows the power factor and Merit Figure composition dependence for the investigated nanocomposite films. Increasing the electrical conductivity enhanced the power factor (PF). The power factor (PF) increased from 2.84 × 10^–5^ (Μw m^–1^ K^–2^) to 2.93 × 10^–3^ (μW m^–1^ K^–2^) for the pure polymer blend and NPFG 25 wt.% films, respectively. Further, the low thermal conductivity, high electrical conductivity, and increasing Seebeck coefficient of the prepared nanocomposite films increased the dimensionless figure of merit by increasing the NPFG content from 2.4 × 10^–8^ to 8.4 × 10^–7^.

for the pure blend and NPFG 25 wt.% films, respectively. The NPFG introduction in the PVA/PANi blend enhances the thermoelectric parameters and sharply increased the Seebeck coefficient value with the increase of the NPFG contents reaching out −50.70 (µV K^−1^) at room temperature, which is higher than that reported by Yihan Wang, et all, where Seebeck coefficient is −20.00 (µV K^−1^) at room temperature for the PANi/GNs corresponds to 32 wt.% GNs content^53^.

## Conclusion

Promising potential of conjugated PVA/PANi blend films doped with functionalized Graphene for thermoelectric applications has been prepared by using a simple in-casting technique. By addressing the challenges in optimizing thermoelectric materials and enhancing their properties through nanostructural engineering, the study contributes to the development of efficient energy-conversion technologies. The ordered molecule structure of the (PVA0.7PANi0.3) polymer chain aligned with the NPFG surface, creating multiple interfaces, and enhancing the electrical and thermoelectrical properties of the prepared films. The increase in NPFG content increases the carrier concentration, carrier mobility, electrical conductivity, thermal conductivity, and the Seebeck coefficient. The maximum electrical conductivity and merit figure were reached for the 25 wt.% NPFG film. It was about two orders of magnitude higher than that of pure polymer blend. The results indicated that the lightweight and low-cost (PVA_0.7_PANi_0.3_)/NPFGx nanocomposite films might be a promising material for thermoelectric applications at the low-temperature range.

## Data Availability

All data supporting this study and its findings are available within the article any data deemed relevant are available from the corresponding author upon request.
